# Laparoscopic Nissen fundoplication is more cost-effective than open Nissen fundoplication in children

**DOI:** 10.1007/s00383-025-05968-1

**Published:** 2025-01-16

**Authors:** Niclas Högberg, Johan Danielson, Amanda Westblom, Elisabet Gustafson

**Affiliations:** 1 Department of Women’s and Children’s Health, Uppsala University, Uppsala, Sweden; 2 Section of Pediatric Surgery, Uppsala University, Akademiska Sjukhuset, 75185 Uppsala, Sweden

**Keywords:** Gastroesophageal reflux, GERD, Laparoscopic, Open, Nissen, Fundoplication, Cost

## Abstract

**Background:**

Nissen fundoplication is one of the most common surgical procedures for gastroesophageal reflux. Current and previous research comparing laparoscopic Nissen fundoplication (LNF) versus open Nissen fundoplication (ONF) in children suggest ambiguous conclusions. The purpose of this retrospective study was to compare the outcome for children operated with LNF or ONF at our institution and to evaluate the economic aspects.

**Methods:**

32 consecutive patients (LNF: *n* = 18, ONF: *n* = 14) operated with Nissen fundoplication between the years 2011–2017 were included in the study. Data was collected by examination of the patient journals and preoperative, operative, postoperative, and post-discharge parameters were analyzed.

**Results:**

Compared to ONF, the LNF group had shorter operating time (165.2 vs 216.6 min, *p* < 0.05), shorter overall operating room duration (315.0 vs 334.9 min, *p* < 0.05) and shorter postoperative hospital stay (3.8 vs 8.1 days, *p* < 0.05). The LNF group also required less morphine (1.2 vs 1.7 mg/kg, *p* < 0.05) and the total cost per case was 39% lower (261.1 vs 427.4 kSEK, *p* < 0.05). No difference was seen in postoperative complications and results.

**Conclusion:**

Laparoscopic Nissen fundoplication is more cost-effective compared to open surgery and reduces postoperative hospital stay and morphine requirements.

## Introduction

### Background

Fundoplication for the treatment of gastroesophageal reflux disease (GERD) was first described by Rudolph Nissen in 1956 1. Since then, several alternative techniques have been proposed, but Nissen’s original open procedure (ONF) is still one of the most widely used methods in surgical practice today. With the evolution of laparoscopy, it became technically feasible to adapt the original technique to a laparoscopic approach. The first paper on laparoscopic Nissen fundoplication (LNF) in adults was published in 1991 2 and the first publication on LNF performed in paediatric surgery soon followed in 1993 3. Several studies of single-centre experiences, as well as randomised studies, have been published on this subject 2, 4–7. The conclusion from these studies varies and the main concern is whether the risk of recurrence of gastroesophageal reflux is higher in patients operated with LNF, which has been reported in some studies 4. Recent reviews on the subject 8, 9 conclude that LNF is an effective surgical alternative to ONF for gastroesophageal reflux in children.

In 2014 we performed our first LNF and in 2016 we decided to use LNF as the standard operative procedure for patients with GERD at our tertiary center for pediatric surgery. In our study, GERD was defined as objectively confirmed gastroesophageal reflux combined with troublesome symptoms and/or complications.

### Aims

The aims of this study were to investigate any differences between LNF and ONF in 1. Operative and total theatre time 2. Length of hospital stay 3. The amount of administered morphine during hospital stay 4. Total costs for surgery and hospital stay 5. Complications and results.

## Materials and methods

### Data collection

The operative registry at the Department of Pediatric Surgery, Uppsala University Hospital, Sweden, was investigated for patients < 18 years of age operated with Nissen fundoplication for GERD between January 2011 and December 2017. A total of 32 patients were included in the study. The data was accessed between January 15th 2018 to May 19th 2018. The authors had access to patient’s charts, and therefore individual patients could be identified during data collection.

### Patient characteristics and indications for surgery

18 patients were operated with LNF (2016–2017) and 14 patients with ONF (2011–2015). The indications for surgery are summarized in Table 1. Each patient could have several indications for surgery. No difference was seen in patient characteristics regarding sex, age, weight or indications for GERD surgery between the two groups (*p* > 0.05). In the LNF group, neurological impairment was present in 72% vs 68% in the ONF group. One patient in the ONF group had previously undergone open repair of esophageal atresia. In the LNF group, one patient was preoperatively diagnosed with gastroparesis on scintigraphy.

**Table 1:** Indications for GERD-surgery

	LNF (*n* = 18)	ONF (*n* = 14)	*p*
Recurrent vomiting; *n* (%)	16 (89%)	11 (79%)	0.38
Aspiration pneumonia; *n* (%)	7 (39%)	5 (36%)	0.57
Esophagitis; *n* (%)	9 (50%)	9 (64%)	0.33
Failure to thrive; *n* (%)	6 (33%)	6 (43%)	0.43
Apnea; *n* (%)	2 (11%)	2 (14%)	1.00

### Preoperative investigation

The preoperative investigations are presented in Table 2, and the pathological findings are presented in Table 3. The preoperative workup was more stringent in the LNF group but there was no difference in the incidence of performed preoperative investigations (*p* > 0.05).

**Table 2:** Performed preoperative investigations

	LNF (*n* = 18)	ONF (*n* = 14)	*p*
Endoscopy (± biopsies); *n* (%)	17 (94%)	11 (79%)	0.30
Esophageal pH monitoring; *n* (%)	17 (94%)	11 (79%)	0.30
Upper GI series; *n* (%)	11 (61%)	10 (71%)	0.71
Gastric emptying scintigraphy; *n* (%)	6 (33%)	3 (21%)	0.69

**Table 3:** Pathological findings in the preoperative investigations

	LNF	ONF	*p*
Endoscopy (± biopsies); *n*/total (%)	9/17 (53%)	9/11 (82%)	0.23
Esophageal pH monitoring; *n*/total (%)	16/17 (94%)	6/11 (55%)	< 0.05
Upper GI series; *n*/total (%)	0/11 (0%)	0/10 (0%)	1.00
	2/6 (33%)	1/3 (33%)	1.00

### Registered perioperative parameters

The perioperative parameters studied were; operating time, total operating room duration, simultaneous operations or procedures, surgeons, and assistants.

### Registered postoperative parameters

The direct postoperative parameters studied were; hospital stay and administered morphine dose per kilogram during the hospital stay. Follow-up parameters/complications that were recorded included retching, dysphagia, GERD symptoms; diagnosed GERD; reoperations and performed dilatations.

### Surgical technique

For laparoscopic procedures, patients were operated in the prone position with the surgeon standing between the legs of the patient. A 5 mm port was introduced in the umbilicus with open technique and pneumoperitoneum was established. Two 5 mm ports were introduced, one to the right of the umbilicus and one in the left flank. A 7–8 mm STEP port was introduced between the port in the left flank and the umbilicus. A 5 mm trocar was then used to make a wound in the epigastrium, through this wound a Nathanson retractor was introduced and used to retract the liver. The camera was introduced in the umbilical port and the assistant used the left lateral port. The head of the table was elevated to facilitate access to the operative area. Any existing gastrostomy was left in place.

For open procedures, patients were operated in the prone position. An upper midline incision was performed, and retractors were applied to the liver and abdominal wall. Any existing gastrostomy was temporarily taken down.

The medial aspect of the gastroesophageal junction (GEJ) was then dissected with electrocautery to visualize the left crus. Dissection was continued on the anterior side of the GEJ and then on the lateral side until the right crus was identified. In ONF, the phrenoesophageal ligament was divided and esophagocrural sutures were placed, whereas in LNF the phrenoesophageal ligament was preserved. Care was taken to identify and avoid damage to the vagal nerves. A retroesophageal window was created, and a cruroplasty was created with two or three 3:0 braided non-absorbable interrupted sutures (Ethibond, Ehicon). The fundus of the stomach was mobilised from the spleen by dividing the short gastric vessels. When the fundus was deemed mobilised enough, the fundus was pulled through the retroesophageal window to perform a 360-degree wrap. Three interrupted, braided non-absorbable 3:0 sutures (Ethibond, Ethicon) were used to create the wrap and fasten it to the anterior part of the esophagus. A fourth suture was put in the anterior part of the wrap and secured to the anterior part of the diaphragm.

For laparoscopic procedures, all ports were taken out under laparoscopic vision and the fascia and subcutis was closed with absorbable sutures. Tissue glue was used to close the skin. For open procedures, incision was closed in layers with absorbable sutures in fascia, subcutis and skin, with a wound catheter placed under the fascia.

### Ethical considerations

The study was approved by the Regional Ethical Committee at Uppsala University, Uppsala, Sweden, approval number 2018/042. The approval did not require individual patient/family consent since the study was based on registered data.

### Statistical methods

Values are presented as proportions, means, medians or range as appropriate. Fisher’s two-tailed exact test was used to compare proportions. The Mann–Whitney *U*-test was used for unpaired comparisons. A *p*-value of less than 0.05 was considered statistically significant. Statistica 13.2 software (Dell, Tulsa, USA) was used for the statistical analysis.

## Results

### Perioperative parameters

**Fig. 1:**
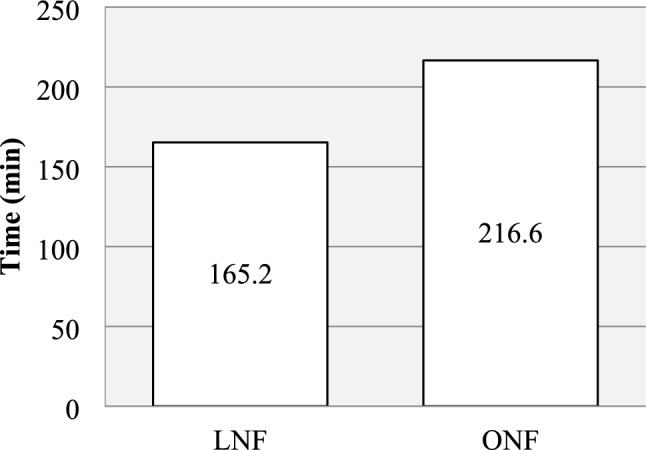
Mean operating time was shorter for LNF (range 66–251) vs ONF (range 121–405), *p* < 0.05

**Fig. 2:**
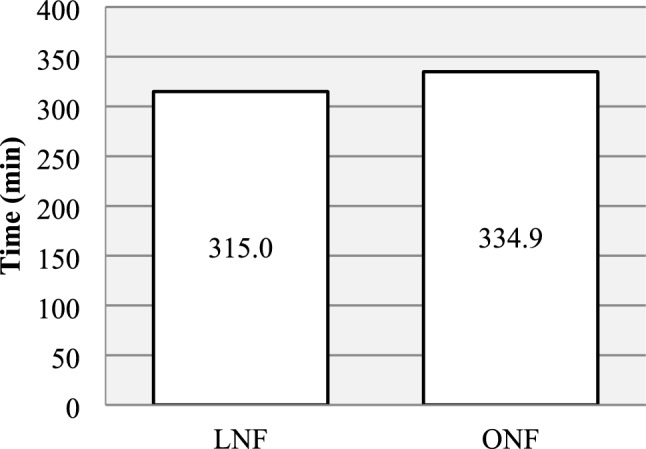
The mean total operating room duration was shorter for the LNF (range 199–545) than ONF (range 226–505), *p* < 0.05

A summary of the perioperative parameters is presented in Figs. 1, 2 and Table 4. Mean operating time was shorter for the LNF group (165 min, SD 71.5) compared to the ONF group (216 min, SD 70.5) (*p* < 0.05). Overall operating room duration was shorter for the LNF group (315 min, SD 90.4) compared to the ONF group (335 min, SD 71.3) (*p* < 0.05). However, more patients in the ONF group underwent simultaneous operations or procedures; 8 patients in the LNF group (44%) and 10 patients in the ONF group (71%), (*p* < 0.05). These included insertion of gastrostomy (*n* = 11), insertion of central venous catheter (*n* = 4), insertion of venous port (*n* = 2), teeth inspection (*n* = 2), incisional hernia repair (*n* = 1), Morgagni-Larrey hernia repair (*n* = 1), adenoidectomy (*n* = 1), Botox injection in peripheral muscles (*n* = 1), loop ileostomy (*n* = 1), removal of jejunostomy (*n* = 1), recanalization of venous port (*n* = 1), change of jejunal tube (*n* = 1) and endoscopic dilation of esophagus (*n* = 1).

**Table 4:** No difference was seen between groups regarding postoperative complications, except GERD symtoms that was reported more frequently in the ONF group

	LNF (*n* = 18)	ONF (*n* = 13)	*P*
Retching; *n* (%) CMIA	4 (22%)	3 (23%)	1.00
Dysphagia; *n* (%) CMIA	6 (33%)	5 (38%)	1.00
GERD symptoms; *n* (%) CMIA	1 (6%)	5 (38%)	0.06
Diagnosed GERD; *n* (%) CM1B	1 (6%)	2 (15%)	0.57
Reoperation; *n* (%) CMIIIB	1 (6%)	0 (0%)	1.00
Dilation; *n* (%) CMIIIA	1 (6%)	1 (8%)	1.00

Complications according to the Clavier-Madadi classification (CM1-IV)

### Postoperative parameters

**Fig. 3:**
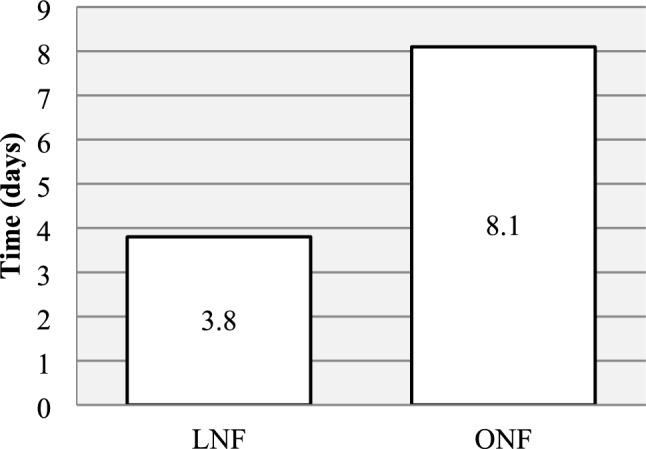
Mean postoperative hospital stay was shorter for LNF (range 1–14) vs ONF (range 6–15), *p* < 0.05

**Fig. 4:**
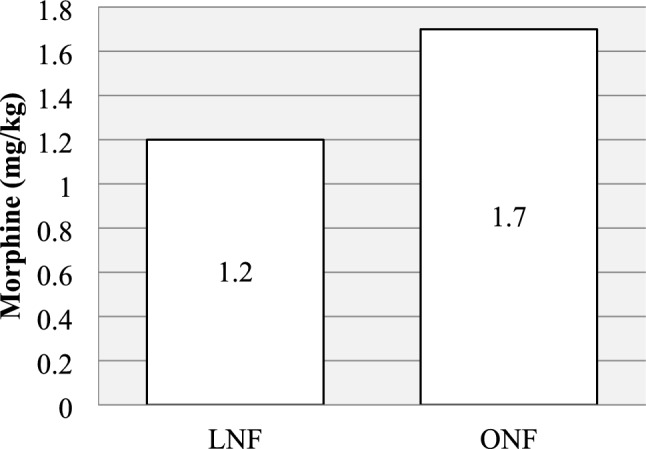
The mean intravenous morphine requirement was lower for the LNF group (range 1.0–2.65) than for the ONF group (range 1.1–3.1), *p* < 0.05

The postoperative parameters are presented in Figs. 3, 4 and Table 4. No difference was seen between the two groups in requirement of intensive care. Mean postoperative hospital stay was 3.8 days (SD 3.2) in the LNF group and 8.1 days (SD 2.54) in the ONF group. The analgesic measures were registered as presence of wound catheter and requirement of intravenous morphine. All patients in the ONF group (100%) and no patient in the LNF group (0%) were given a wound catheter. Mean morphine requirement was lower in the LNF group compared to the ONF group (1.2 mg/kg (SD 0.4) vs. 1.7 mg/kg (SD 0.61) (*p* < 0.05). Two patients in the LNF group (11%) and one patient in the ONF group (7%) suffered from postoperative infection (*p* > 0.05).

### Complications and follow-up

All patients were followed-up either by return visit, phone call, video conference or a combination of methods within six months postoperatively. Continued follow-up was thereafter based on clinical symptoms. One patient in the ONF group died 6 months after the operation, unrelated to the fundoplication. This patient was therefore not available for follow-up and is excluded from these results.

The postoperative complications are presented in Table 4. No difference was seen regarding retching, dysphagia, or need for dilatation or redo-fundoplication. No difference was seen between the groups regarding the recurrence of GERD.

### Economical aspects, total cost

**Fig. 5:**
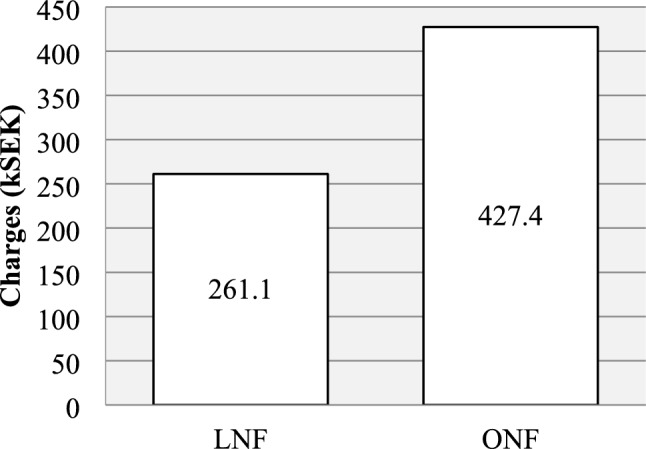
Mean total hospital cost was 39% lower for LNF (range 100.5–747.4) vs ONF (range 256.3–666.9), *p* < 0.05

The total hospital charges are presented in Fig. 5. For each patient, the total cost was calculated based on the local billing charges for the operation (operating time, anesthesia, surgical material, wound catheter) and hospital stay (days in hospital, time in recovery room, admittance at an intensive care unit). Mean total costs were 261.1 kSEK (SD 171.9, approximately 26.1 kEuro) in the LNF group and 427.4 kSEK (SD 137.8, approximately 42.74 kEuro) in the ONF group. Mean cost was 166.3 kSEK (approximately 16.63 kEuro) or 39% lower for LNF than for ONF (*p* < 0.05).

## Discussion

This study has several interesting findings. Compared to ONF, LNF shortened the mean operating time and the mean overall operating room duration. The LNF group also required less morphine and dramatically shortened the mean hospital stay duration by more than four days. Furthermore, the total cost for LNF was 39% lower while no difference was seen in postoperative results between the two groups.

Three randomized trials comparing LNF and ONF in children have previously been published 6, 7, 10. All of them found that LNF was associated with significantly longer operating time than ONF. The study by Papandria et al. 10 also declared that the LNF group had longer overall operating room duration. In our study, however, laparoscopy was found to shorten both the operating time and the overall operating room duration. In the previously published studies, the mean operating times were 160 min 7, 150 min 6 and 173 min 10 in the LNF group, and 80 min 7 89 min 6 and 91 min 10 in the ONF group. Thus, our mean operating time for the LNF group was in line with the operating times in the previously published studies, although our data includes a learning curve for performing surgeons, as the LNF technique was introduced at our centre during this study. However, our mean operating time for the ONF group was substantially longer than reported in the previous studies.

ONF, however, had been performed for several years before the first patients in our study were operated, and consequently two of the surgeons were already well experienced with the operative technique. The third surgeon started performing ONFs during the study period, wherefore that learning curve is included in the ONF group. Nevertheless, it should be noted that all surgeons performing the LNFs in our study were already experienced laparoscopists when they started with the LNFs. The previous laparoscopic experience among the surgeons might explain our short operating times, despite the included learning curve for LNF. One factor that possibly could affect our results is that four different surgeons performed the operations. Consequently, the surgeons’ individual skills and rapidity could cause the variation in time. On the other hand, two of the four surgeons performed both the ONFs and the LNFs, hence the individual factor should not be the only cause for the difference in operating time and overall operating room duration. Furthermore, different surgeons have also performed the fundoplications in the three previous studies. This additionally confirms the theory that the individual skill of the surgeon should not affect the results to a great extent.

Moreover, another important factor to consider is that our study was retrospective. This caused difficulty in identifying the correct operating times and overall operating room durations, especially for the patients who underwent simultaneous operations or procedures in connection with the Nissen fundoplication. This was particularly difficult for the operations executed before the autumn of 2016 since a new electronic system for registration of surgery was introduced at our hospital. Consequently, this would mainly affect the operating times and overall operating room durations for the ONF group since these operations were all executed before 2016 and 71% of the patients in the ONF group underwent simultaneous operations or procedures, in comparison with 44% in the LNF group. Thus, this could contribute to the difference in operating time and overall operating room duration between the ONF and LNF group in our study.

It is well-recognized among surgeons that laparoscopic surgery in general is associated with shorter postoperative hospital stay 11. In this study, LNF shortened the hospital stay by 4.3 days compared to ONF. In the previous publications, the median hospital stay was 5.0, 7.0, and 6.0 days, respectively, for the LNF group and 4.5, 7.5, and 4.0 days for the ONF group 6, 7, 10. Accordingly, our study is from what we know the only study demonstrating a dramatically shorter hospital stay for LNF, due to a more rapid patient recovery.

Our study also demonstrates decreased costs for the laparoscopy group. Previous studies 6, 7 did not analyze the costs of GERD surgery. One study found higher operating room costs for LNF, but no difference in total hospital charges between the groups 10.

The main reason for the distinct difference in mean total hospital charges between the two groups in our study is due to the shorter hospital stay for the LNF group. It was also a slightly higher proportion of patients who needed intensive care after ONF, which also increased the costs for that group. Even though the surgical material and equipment were more expensive for LNF, this difference was negligible in the full cost summation.

In contrast to the previous randomized trials 7, 10, LNF significantly decreased the mean morphine requirements in our study. Hence, our study seems to be the first study published during the last decade that has registered less use of morphine after LNF. It is well-recognized among surgeons that laparoscopy in general is associated with less postoperative pain 12.

In terms of complications after the operation, the previously published studies have presented ambiguous results. Retching was significantly more common after ONF 7, and the difference still remained after four years in the follow-up study 13. No difference was seen in early complications between the groups 6, 10, whereas a follow-up study 4 registered both a higher recurrence rate of GERD and a higher rate of reoperation after LNF. As to our study, no significant difference was seen between the groups in the incidence of both postoperative and post-discharge complications. Postoperative investigation was only performed if the patient showed symptoms of recurrence of GERD or was part of a follow-up program for another medical condition (for example long gap esophageal atresia). Only one patient in the LNF group suffered from symptoms of GERD and this patient was also diagnosed with recurrence and re-operated six months after the initial Nissen fundoplication. Of the patients suffering from symptoms of GERD in the ONF group, only one patient was diagnosed with a recurrence of GERD by pH-monitoring.

## Conclusion

The present study clearly indicates the advantages of LNF over ONF: shorter operating time, shorter total hospital stay and faster recovery, less morphine requirements and lower overall cost, at equal postoperative outcomes. Our results clearly indicates that laparoscopy should be the preferred technique for Nissen fundoplication in children.

## Data Availability

No datasets were generated or analysed during the current study.
